# *Tetranychus* *evansi* spider mite populations suppress tomato defenses to varying degrees

**DOI:** 10.1002/ece3.6204

**Published:** 2020-04-12

**Authors:** Bram Knegt, Tomas T. Meijer, Merijn R. Kant, E. Toby Kiers, Martijn Egas

**Affiliations:** ^1^ Department of Evolutionary and Population Biology Institute for Biodiversity and Ecosystem Dynamics University of Amsterdam Amsterdam The Netherlands; ^2^ Department of Ecological Science VU University Amsterdam The Netherlands

**Keywords:** biotic interactions, herbivore offense, intraspecific variation, jasmonate reporter, Plant–herbivore interactions, *Solanum lycopersicum*

## Abstract

Plant defense suppression is an offensive strategy of herbivores, in which they manipulate plant physiological processes to increase their performance. Paradoxically, defense suppression does not always benefit the defense‐suppressing herbivores, because lowered plant defenses can also enhance the performance of competing herbivores and can expose herbivores to increased predation. Suppression of plant defense may therefore entail considerable ecological costs depending on the presence of competitors and natural enemies in a community. Hence, we hypothesize that the optimal magnitude of suppression differs among locations. To investigate this, we studied defense suppression across populations of *Tetranychus evansi* spider mites, a herbivore from South America that is an invasive pest of solanaceous plants including cultivated tomato, *Solanum lycopersicum*, in other parts of the world. We measured the level of expression of defense marker genes in tomato plants after infestation with mites from eleven different *T. evansi* populations. These populations were chosen across a range of native (South American) and non‐native (other continents) environments and from different host plant species. We found significant variation at three out of four defense marker genes, demonstrating that *T. evansi* populations suppress jasmonic acid‐ and salicylic acid‐dependent plant signaling pathways to varying degrees. While we found no indication that this variation in defense suppression was explained by differences in host plant species, invasive populations tended to suppress plant defense to a smaller extent than native populations. This may reflect either the genetic lineage of *T. evansi*—as all invasive populations we studied belong to one linage and both native populations to another—or the absence of specialized natural enemies in invasive *T. evansi* populations.

## Graphical abstract

Suppression of plant defenses by herbivores promotes herbivore performance, but may also increase competition and predation. We hypothesize that biotic interactions with competitors and natural enemies shape the costs and benefits of defense suppression, and found significant variation in the magnitude of defense suppression among eleven populations of the defense‐suppressing spider mite *Tetranychus evansi*, ranging from Brazil to Japan. Invasive populations tended to suppress plant defense to a smaller extent than native populations, and we speculate that this may reflect the absence of specialized natural enemies in invasive *T. evansi* populations.

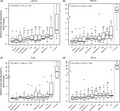

## 1 INTRODUCTION

Plants and herbivores share a 420 million year history of antagonistic coevolution (Labandeira, 1998). Over this time, these adversaries have been in an arms race of adaptations and counter‐adaptations. This has resulted in the evolution of elaborate plant defense mechanisms, such as two‐component toxins (Matile, 1980) and recruitment of natural enemies with plant volatiles (Baldwin & Schultz, 1983; Heil, 2014). In response, herbivores have evolved offensive traits that enable them to consume plant tissues more efficiently, such as mechanisms to detoxify defensive plant compounds (Heckel, 2014; Smith, 1955). Over the last decade, herbivores were also found to suppress plant defense by manipulating plant physiological processes, thereby promoting herbivore performance (Kant et al., 2015; Musser et al., 2002). Whiteflies, for example, normally induce a defense response in their host plants that is regulated by the plant hormone jasmonic acid (JA; van de Ven, LeVesque, Perring, & Walling, 2000; Walling, 2000). *Bemisia tabaci* silverleaf whiteflies, however, hijack defense regulation of their *Arabidopsis thaliana* hosts by inducing salicylic acid (SA)‐dependent defense signaling (Zarate, Kempema, & Walling, 2007). Induced SA levels suppress JA levels through hormonal cross talk (Thaler, Humphrey, & Whiteman, 2012) and hence protect silverleaf whiteflies from JA‐dependent defenses. More than twenty arthropod herbivore species suppress plant defenses (Kant et al., 2015), and a majority are crop pest species, such as the corn earworm (*Helicoverpa zea*; Musser et al., 2002), the Colorado potato beetle (*Leptinotarsa decemlineata*; Lawrence, Novak, & Blackburn, 2007), and the spider mites *Tetranychus urticae* (Kant, Sabelis, Haring, & Schuurink, 2008) and *Tetranychus evansi* (Alba et al., 2015; Sarmento, Lemos, Bleeker, et al., 2011).

Understanding why defense suppression is a successful herbivore offense strategy requires insight into its evolutionary costs and benefits (Blaazer et al., 2018). A benefit of defense suppression for herbivores is that it prevents expression of plant defense, which would otherwise have resulted in reduced herbivore performance (Kant et al., 2015; Musser et al., 2002). At the same time, however, defense suppression creates a hospitable, nutritious plant (i.e., a “public good,” Rankin, Bargum, & Kokko, 2007) from which competitors and natural enemies can also benefit (Alba, Glas, Schimmel, & Kant, 2011; Ataide et al., 2016; Kant et al., 2015). Such biotic interactions introduce new costs. A prime example of such costs can be found in *Tetranychus evansi* spider mites (Blaazer et al., 2018). By suppressing tomato defense, *T. evansi* increase not only their own performance but also that of competing *Tetranychus spp.* spider mites (Alba et al., 2015; Godinho, Janssen, Dias, Cruz, & Magalhães, 2016; Sarmento, Lemos, Bleeker, et al., 2011). In addition, defense suppression by *T. evansi* exposes their offspring to increased predation by *Phytoseiulus longipes* predatory mites, possibly due to reduced transfer of defensive compounds from plants through spider mites into their eggs (Ataide et al., 2016). Defense suppression may therefore entail considerable costs depending on the biotic environment in which it is employed (Glas et al., 2014; Schimmel, Ataide, Chafi, et al., 2017).

To assess the role of biotic interactions in the evolution of defense suppression, it is pivotal to quantify variation in defense suppression across different biotic environments. In *Leptopilina boulardi* parasitoid wasps, for example, genotypes from different localities were found to suppress the immune system of their *Drosophila* hosts to varying degrees, depending on the abundance of specific host species (Dupas & Boscaro, 1999). Yet, in herbivores, variation in defense suppression has hitherto been investigated only scarcely (Alba et al., 2015). Here, we aim to quantify variation in the magnitude of suppression of plant defense among populations of the defense‐suppressing spider mite *T. evansi* and secondarily to explore whether differences relate to characteristics of their biotic environments. Specifically, we assessed if variation was explained by the host plant from which populations were sampled and by the presence or absence of specialized natural enemies from native and non‐native environments.

The tomato red spider mite *T. evansi* Baker and Pritchard (Acari: Tetranychidae) is a herbivorous spider mite from South America, feeding mainly from solanaceous host plants such as tomato, potato, and eggplant (Migeon & Dorkeld, 2018; Navajas, Moraes, Auger, & Migeon, 2013). Spider mites are cell content‐feeders, piercing plant parenchyma cells with their stylets, sucking up the contents, and leaving behind empty cells that are visible as white feeding scars (Bensoussan et al., 2016). *T. evansi* occurs mostly in tropical, subtropical, and Mediterranean climates, and can reach fast intrinsic rates of population increase due to its short generation time (<15 days), especially at high temperatures (Bonato, 1999; Gotoh et al., 2010). Over the last twenty years, *T. evansi* has become invasive in many areas with subtropical and Mediterranean climates, such as sub‐Sahara Africa, the Mediterranean region, and East Asia (Navajas et al., 2013). In its invasive range, *T. evansi* replaces *T. urticae* and other indigenous spider mite species as the dominant species in spider mite communities and colonizes new host plant species (Azandémè‐Hounmalon et al., 2015; Ferragut, Garzón‐Luque, & Pekas, 2013).

The mechanism of defense suppression by *T. evansi* has mostly been studied on cultivated tomato plants, *Solanum lycopersicum*. In tomato, the induced defense response against spider mites is orchestrated by the plant hormones JA and SA (Ament, Kant, Sabelis, Haring, & Schuurink, 2004; Kant, Ament, Sabelis, Haring, & Schuurink, 2004; Li, Williams, Loh, Lee, & Howe, 2002). These hormones set in motion internal signaling cascades leading to the production of defense‐associated compounds such as proteinase inhibitors (PIs) and polyphenol oxidases (PPO; Arnaiz et al., 2018; Kant et al., 2004; Martel et al., 2015). *T. evansi* suppress tomato defense downstream of plant hormone accumulation, such that the plant's expression of defense‐associated genes is downregulated to the benefit of the herbivore (Alba et al., 2015; Ataide et al., 2016; Sarmento, Lemos, Bleeker, et al., 2011). In the course of the infestation, such suppression can temporarily result in expression levels of defense genes at or below the plant's housekeeping levels (Sarmento, Lemos, Bleeker, et al., 2011) but for most of the time in an intermediate level of induction (Alba et al., 2015; Schimmel et al., 2018). This suppression is mediated by secreted salivary effector proteins (Jonckheere et al., 2016; Villarroel et al., 2016) that restrain the defense response to levels of induction low enough for the mite to tolerate (Ataide et al., 2016), independent of herbivore‐associated bacteria (Staudacher et al., 2017). Expression levels of defense‐associated plant genes therefore are an adequate measure of defense suppression by *T. evansi*, as long as timing is standardized and a benchmark treatment for defense induction is included (Alba et al., 2015; Sarmento, Lemos, Bleeker, et al., 2011; Schimmel, Ataide, Chafi, et al., 2017).

Plant identity and diversity affect interactions between plants and herbivores (Agrawal, Lau, & Hambäck, 2006). Arthropod communities, for example, differ among locations due to variation in secondary metabolites of their host plants (Bálint et al., 2016; Bangert et al., 2006; Glassmire et al., 2016; Poelman, Loon, & Dicke, 2008; Randlkofer, Obermaier, Hilker, & Meiners, 2010; Richards et al., 2015). The host range of *T. evansi* includes more than a hundred plant species, mainly from the Solanaceae family (Migeon & Dorkeld, 2018), with considerable variation in secondary metabolites and resistance to herbivory (Fridman et al., 2005; Spooner, Peralta, & Knapp, 2005; Wink, 2003). The *T. evansi* populations investigated in this study were sampled from four host plant species, all belonging to the *Solanum* genus (Table 1). *Solanum* species produce different levels of glycoalkaloids and proteinase inhibitors that differentially affect herbivore performance, are therefore likely to harbor different arthropod communities (Cipollini & Levey, 1997; Girard et al., 2007; Hartl, Giri, Kaur, & Baldwin, 2010; Jared, Murungi, Wesonga, & Torto, 2016; Nohara et al., 2007; Tingey, 1984), and may expose defense‐suppressing herbivores to different levels of competition and predation. Consequently, we explored if the level of defense suppression by *T. evansi* varied among populations collected from these host plants.

**Table 1:** Collection records of *Tetranychus evansi* populations used in this study

Population	Laboratory host	Field host	Location	Range	Latitude	Longitude	Date	Collector	Reference
Algarrobo‐1	*S. lycopersicum* cv. Castlemart	*S. nigrum*	Andalucía, Spain	invasive	36°45′N	4°02′W	2011	J.M. Alba	Alba et al. (2015)
Carangola‐1	*S. lycopersicum* cv. Santa Clara	*S. lycopersicum*	Minas Gerais, Brazil	native	20°44′S	42°02′W	2013	J. Mencalha	This study
Chiyoda‐1	*S. nigrum*	*S. nigrum*	Tokyo, Japan	invasive	35°40′N	139°45′E	Sep 2010	T. Gotoh	This study
JT	*S. nigrum*	*S. nigrum*	Tokyo, Japan	invasive	35°35′N	139°36′E	Nov 2006	T. Gotoh, Y. Kitashima	Gotoh et al. (2009)
Kagoshima‐1	*S. nigrum*	*S. nigrum*	Kagoshima, Japan	invasive	31°34′N	130°30′E	Jul 2009	Y. Sakamaki	Ikeshima et al. (2009)
KM	*S. nigrum*	*S. lycopersicum*	Makueni County, Kenya	invasive	01°42′S	37°25′E	Mar 2001	M. Knapp	Gotoh et al. (2009)
SC	*S. nigrum*	*S. lycopersicum*	Canary Islands, Spain	invasive	28°23′N	16°33′W	Dec 2006	E. Hernandez‐Suarez	Gotoh et al. (2009)
Sde Eliyahu‐1	*S. lycopersicum defenseless‐1* ^b^	*S. tuberosum* and *S. melongena*	Mo'atza Azorit Emeq Hamaayanot, Israel	invasive	32°26′N	35°30′E	Jun 2013	A. Tabic	This study
SV	*S. nigrum*	*S. lycopersicum*	Valencia, Spain	invasive	39°29′N	0°20′W	Jan 2007	F. Ferragut	Gotoh et al. (2009)
TW	*S. nigrum*	*S. nigrum*	Wufeng, Taiwan	invasive	24°04′N	121°42′E	Dec 2006	C.‐C. Ho	Gotoh et al. (2009)
Viçosa‐1^a^	*S. lycopersicum* cv. Castlemart	*S. lycopersicum*	Minas Gerais, Brazil	native	20°45′S	42°52′W	2002	A. Pallini	Sarmento, Venzon, Pallini, Oliveira, and Janssen (2007)

^a^ This population was referred to as “BP” by Gotoh et al. (2009, 2010), and as “Vicoça‐1” by Alba et al. (2015). We choose to use the latter name, because it was first collected in Viçosa and described by Sarmento et al. (2007).

^b^ Population Sde Eliyahu‐1 was collected from potato (*S. tuberosum*) and eggplant (*S. melongena*) in a field where also tomato was grown, but none of the tomato plants were infested with *T. evansi*. We reasoned that this lack of preference for tomato could potentially be caused by a different defense suppression phenotype, which we preferred not to select against in laboratory cultures. However, we did not have potato or eggplant leaves available at the time this population arrived, and therefore chose the tomato mutant *defenseless‐1* (*def‐1*), which does not accumulate JA after spider mite feeding (Li et al., 2002), as a host. If the lack of preference for tomato in this population was caused by an inability to suppress JA‐dependent tomato defense, then we prevented selection for more potent suppressors by using *def‐1* host plants.

We also explored if the level of defense suppression differed between native (South American) and invasive (other continents) *T. evansi* populations. The predatory mite *Phytoseiulus longipes* and the entomopathogenic fungus *Neozygites floridiana* are able to severely reduce *T. evansi* populations in their native range (Ribeiro, Gondim, Calderan, & Delalibera, 2009; da Silva et al., 2010), but are absent in areas where *T. evansi* is invasive (Ferragut et al., 2013). Defense suppression by *T. evansi* entails costs in the presence of natural enemies, such as increased egg predation by *P. longipes* (Ataide et al., 2016). The lack of natural enemies in areas where *T. evansi* is invasive may therefore reduce such ecological costs, allowing *T. evansi* to suppress plant defense more strongly as an evolutionary consequence of reduced antagonistic pleiotropy (Cooper & Lenski, 2000).

**Figure 1:**
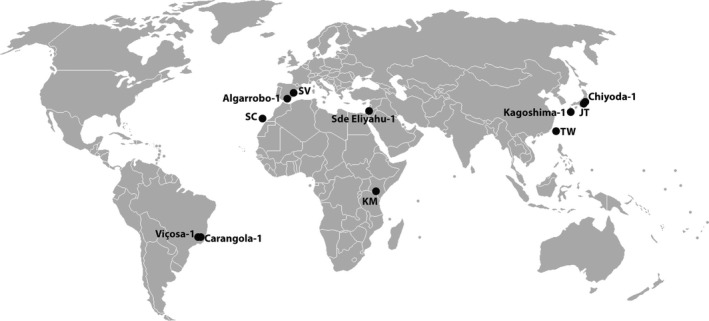
Sampling locations of *Tetranychus evansi* populations used in this study

We sampled *T. evansi* mites from eleven locations across its native and invasive range (Figure 1), and measured their magnitude of defense suppression with two approaches. First, we measured the expression of a reporter gene for defense induction in *pLAP‐A1:GUS* tomato plants. In these plants, the promoter of the *β‐glucuronidase* (GUS) reporter gene is fused to the JA‐dependent promoter of the plant defense‐associated gene *leucine aminopeptidase A1* (*LAP‐A1*; Chao, Gu, Pautot, Bray, & Walling, 1999). When plant defense is induced, *LAP‐A1* is activated, and thereby also the GUS reporter, of which its activity can be determined in a fluorimetric assay (Jefferson, Kavanagh, & Bevan, 1987). Because these assays were more variable than expected, we also measured the level of gene expression the defense‐associated tomato genes *LAP‐A1*, *polyphenol oxidase‐D* (*PPO‐D*), *proteinase inhibitor IIc* (*PI‐IIc*), and *pathogenesis‐related protein 1a* (*PR‐1a*) through quantitative reverse transcription–polymerase chain reaction (qRT‐PCR). *T. evansi* suppresses these genes in tomato (Alba et al., 2015; Sarmento, Lemos, Bleeker, et al., 2011; Schimmel, Ataide, Chafi, et al., 2017). In all experiments, we included a treatment where tomato plants were infested with a defense‐inducing *T. urticae* genotype as a benchmark for defense induction. For those genes where we observed different levels of expression among populations, we investigated if this variation was affected by host plant species or geographical range (invasive or native). We also verified that variation was not due to the identity of their host plants in the laboratory, or the time that populations had been maintained there. Last, to investigate the possibility that differences in suppression could be explained by differences in genetic lineage (Boubou et al., 2012; Gotoh et al., 2009), we sequenced a part of the mitochondrial *cytochrome oxidase subunit 1* gene (CO1).

## 2 MATERIALS AND METHODS

### 2.1 Spider mite populations and culture conditions

We obtained eleven *T. evansi* populations that had been collected by other research groups from several locations across South America, Europe, Africa, and Asia (Figure 1, Table 1). Because a change in host plant can have drastic consequences for genetic and phenotypic variation within a population (Dermauw et al., 2013; Magalhães, Blanchet, Egas, & Olivieri, 2009; Wybouw et al., 2015), we maintained them on the same host plant as they had been on in the research group from which we obtained these populations, that is, ached *S. nigrum* leaves or *S. lycopersicum* leaflets. We placed leaves and leaflets with their abaxial side facing upwards on wet cotton wool in open plastic trays in a controlled environment (25°C; 16:8 hr light: dark photoperiod; 60% relative humidity). We grew plants in a greenhouse (25:18°C; 16:8 hr photoperiod; 50%–60% relative humidity) for 4–5 weeks before leaves were used to feed the mite cultures.

### 2.2 Infestation treatments and sampling

We obtained *pLAP‐A1:GUS* seeds from Linda Walling (University of California, Riverside, USA). In these plants, the promoter of the *β‐glucuronidase* (GUS) reporter gene is fused to the JA‐dependent promoter of the plant defense‐associated gene *leucine aminopeptidase A1* (*LAP‐A1*; Chao et al., 1999). When plant defense is induced, *LAP‐A1* is activated, and thereby also the GUS reporter, of which its activity can be determined in a fluorimetric assay (Jefferson et al., 1987). We grew *pLAP‐A1:GUS* and untransformed UC82 tomato plants in a greenhouse (25:18°C; 16:8 hr photoperiod; 50%–60% relative humidity) for 11–14 days and then transferred them to a climate room (25°C; 16:8 hr light: dark photoperiod; 60% relative humidity) to acclimatize for 7–10 days, such that plants were exactly 21 days old at the start of the experiments*. *We only used plants with three or four expanded leaves and included this difference as a variable in our analyses (“plant stage,” see below). We infested *pLAP‐A1:GUS* plants with 45 age‐synchronized (14 days after oviposition and thus 2–4 days old) *T. evansi* females for 1 day, by manually transferring individual mites with a fine brush to three leaflets (second, third, or terminal) of three different leaves per plant, such that each leaflet received 15 mites. We prepared a lanolin barrier around the petiole at the base of each infested leaflet to confine mites to the infested leaflets. We included a benchmark treatment for defense induction by infesting *pLAP‐A1:GUS* plants with mites from a defense‐inducing *T. urticae* genotype (previously called “KMB” in Kant et al., 2008, renamed to “Santpoort‐2” by Alba et al., 2015), as well as uninfested *pLAP‐A1:GUS* plants and uninfested, untransformed UC82 plants as negative controls. Uninfested *pLAP‐A1:GUS* and UC82 control plants also received lanolin, as well as a mock infestation through gently touching leaflets with a clean brush.

Because a pilot experiment indicated that differences among suppression and induction benchmarks for GUS activity were most pronounced after 1 day of infestation, we harvested infested leaflets after 1 day. We digitally scanned them (HP Scanjet G3110, Hewlett‐Packard) to determine leaf damage (see next section) and flash‐froze them within 2 min after harvest in 15 ml tubes in liquid nitrogen for storage at −80°C. We performed the experiments in five blocks in time, such that all 14 treatments, 11 T*. evansi* populations, the induction benchmark treatment, plus 2 controls, had a sample size of 10–15 plants evenly distributed across blocks (Table 2).

**Table 2:** Treatment details and sample size

Treatment	Mite species	Mite population	Plant genotype	Sample size
Algarrobo‐1	*Tetranychus evansi*	Algarrobo‐1	*pLAP‐A1:GUS*	10
Carangola‐1	*Tetranychus evansi*	Carangola‐1	*pLAP‐A1:GUS*	11
Chiyoda‐1	*Tetranychus evansi*	Chiyoda‐1	*pLAP‐A1:GUS*	12
JT	*Tetranychus evansi*	JT	*pLAP‐A1:GUS*	12
Kagoshima‐1	*Tetranychus evansi*	Kagoshima‐1	*pLAP‐A1:GUS*	12
KM	*Tetranychus evansi*	KM	*pLAP‐A1:GUS*	10
SC	*Tetranychus evansi*	SC	*pLAP‐A1:GUS*	10
Sde Eliyahu‐1	*Tetranychus evansi*	Sde Eliyahu‐1	*pLAP‐A1:GUS*	12
SV	*Tetranychus evansi*	SV	*pLAP‐A1:GUS*	11
TW	*Tetranychus evansi*	TW	*pLAP‐A1:GUS*	12
Viçosa‐1	*Tetranychus evansi*	Viçosa‐1	*pLAP‐A1:GUS*	11
*T. urticae*	*Tetranychus urticae*	Santpoort‐2	*pLAP‐A1:GUS*	12
Control	—	—	*pLAP‐A1:GUS*	11
UC82	—	—	UC82	15

### 2.3 Leaf damage quantification

We quantified leaf chlorotic spots as measure of leaf damage. Even though these spots contain not only cells that collapsed after they were emptied by the mite but also neighboring cells that collapsed later without being eaten, they are routinely used as a proxy for mite feeding intensity (Bensoussan et al., 2016). We quantified the damaged area of each infested leaflet using ImageJ version 1.49 (Rasband, 2016). We transformed RGB‐colored scans of damaged leaflets to black and white images using the Type tool and distinguished damaged from nondamaged leaf area by applying a color threshold typical for spider mite leaf damage using the Adjust Threshold tool. After this step, leaf damage appears as black spots while undamaged leaf surface was white. The background was automatically transformed to dark and ignored during the measurements. We then selected the damaged area within the leaf with the Selection tool and measured damaged leaf area in mm^2^ by using the Analyze Particles tool. We averaged leaf areas across the three damaged leaflets into one value per plant*. *Each scan included a piece of millimeter paper to ensure accurate scaling of leaf size and damaged surface area.

Plants usually respond in a dose‐dependent manner to spider mite damage (Gols, Roosjen, Dijkman, & Dicke, 2003; Horiuchi et al., 2003) and herbivory in general (Agrawal, 2004; Niinemets, Kännaste, & Copolovici, 2013). Therefore, we normalized our measurements of tomato gene expression (GUS assays and qRT‐PCR measurements) to the absolute amount of leaf damage, to correct for variation due to differences in damaged tissue. Normalization is appropriate, because previous work showed that defense gene induction correlates with damage (Alba et al., 2015); note that as a result of the plant's wound response (Wasternack et al., 2006), induction of jasmonate defenses progresses with mite feeding damage (Kant et al., 2004). We also present non‐normalized averages to allow comparison to the uninfested control treatments which have no feeding damage.

### 2.4 Protein extraction and total protein quantification

We ground frozen leaf material in 15 ml tubes by vortexing for 15 s while using two slim metal rods to crush the leaflets. We repeated this step four times. We then transferred leaf material to 2‐ml Eppendorf tubes and manually ground it to fine powder using a sterile pestle for 15 s and repeated manual grinding three times. During both grinding methods, we kept our samples frozen and afterward stored them at −80°C. We extracted total protein by adding 300 µl extraction buffer (50 mM NaPO_4_ (pH 7.2), 1 mM EDTA, 0.1% v/v Triton x‐100 and 0.1% v/v Sarcosyl) to each tube, mixed the samples with a sterile pestle for 10–15 s, and then centrifuged them at 4°C and 17,382 *g* for 2 min. We transferred 200 µl of the protein‐rich supernatant to new Eppendorf tubes and stored these at −80°C. To assess the total amount of protein extracted from each plant tissue sample, we transferred 199 µl miliQ water to a 96‐well plate, after which we added 1 µl protein extract. We then added 50 µl Bio‐Rad protein dye concentrate (Bio‐Rad GmbH) and mixed samples carefully in the tip of a pipette. We added calibration curves samples, containing 0, 0.1, 0.3, 0.5, and 0.7 mg/ml bovine serum albumin (Sigma‐Aldrich, St*. *Louis, USA) and then incubated the plate for 2 min at room temperature after which we measured absorbance at 595 nm using a plate reader (Tecan infinite F50, Tecan Group).

### 2.5 GUS activity assay

*pLAP‐A1:GUS* tomato plants have the *GUS* gene fused to a copy of the promoter (and part of the 5′ untranslated region) of the endogenous *LAP‐A1* gene, such that when the endogenous *LAP‐A1* is expressed, *GUS* enzyme is produced in parallel (Chao et al., 1999). Because (young) tomato plants have no intrinsic GUS activity (Hu et al., 1990), the amount of GUS activity in *pLAP‐A1:GUS* plants is proportional to the expression of the endogenous *LAP‐A1* gene. As a glycosidase, GUS catalyzes the breakdown of carbohydrates. GUS activity can therefore be determined in a fluorimetric assay where nonfluorescent 4‐methylumbelliferyl‐β‐d‐glucuronide (MUG) is transformed by GUS into fluorescent 4‐methylumbelliferone (MU; Jefferson et al., 1987). We transferred 25 µl protein‐rich plant extract to 96‐well microtiter plates, after which we added 25 µl reaction buffer (1 mM MUG, 20 mM β‐mercaptoethanol) and mixed the samples in the tip of a pipette. We covered the microtiter plate with saran wrap and incubated it at 37°C for 90 min. We then added 50 µl stop buffer (0.2 M Na_2_CO_3_·10H_2_0) to stop the reaction and added our calibration curve samples (0.0, 0.05, 0.1, 0.15, 0.2, and 0.3 mM MU) to the plate. We measured fluorescence with a plate reader (BioTek synergy MX, BioTek Instruments) at wavelengths of 360 nm (excitation) and 460 nm (emission).

### 2.6 RNA extraction and cDNA synthesis

Of all treatments (Table 2), we extracted total plant RNA from ground, frozen leaf tissue using the hot phenol method of Verwoerd, Dekker, and Hoekema (1989). We diluted RNA samples such that they reached the concentration of the lowest and then performed a DNAse treatment using an Ambion TURBO DNAse kit (Thermo Fisher Scientific). Briefly, we added DNAse mastermix (2.0 μl 10× DNAse buffer and 0.5 μl DNAse) to 17.5 μl RNA solution, incubated the tubes at 37°C for 40 min, added 2 μl DNAse inactivation reagent, mixed the samples gently at room temperature for 5 min, centrifuged them at 17,382 *g* for 5 min, and then transferred 12.5 μl of the supernatant to new tubes. Next, we synthesized cDNA using a RevertAid First Strand cDNA Synthesis Kit (Thermo Fisher Scientific). We first added 1 μl oligo (dT)_18_ primer and incubated samples at 70°C for 5 min. Then, we added 6.5 μl reverse transcriptase (RT) mastermix (4.0 μl 5× RT buffer, 2.0 μl dNTPs, 0.5 μl RT), synthesized cDNA at 42°C for 60 min, and inactivated the RT enzyme at 70°C for 10 min. We diluted the resulting cDNA solutions 5 times.

### 2.7 Gene expression assays (qRT‐PCR)

To investigate the degree to which the *T. evansi* populations suppressed tomato defense, we measured expression of the defense‐associated tomato genes *LAP‐A1*, *polyphenol oxidase‐D* (*PPO‐D*), *proteinase inhibitor IIc* (*PI‐IIc*), and *pathogenesis‐related protein 1a* (*PR‐1a*) in all treatments (Table 2). We used the tomato *actin* gene as a housekeeping reference (Løvdal & Lillo, 2009). Expression of *LAP‐A1* was demonstrated to depend on JA defense signaling (Chao et al., 1999), and JA‐dependent regulation is likely for *PPO‐D* and *PI‐IIc*, since tomato JA accumulation mutants have no *polyphenol oxidase‐F* or any *PI‐II* expression (Li et al., 2004). *PR‐1a* is associated with SA signaling, because tomato *PR1a* expression increases upon exogenous application of SA (van Kan, Cozijnsen, Danhash, & Wit, 1995), and tomato *PR‐1a* is highly similar to *PR‐1a* in tobacco (van Kan, Joosten, Wagemakers, Berg‐Velthuis, & Wit, 1992), which is regulated by SA (Niki, Mitsuhara, Seo, Ohtsubo, & Ohashi, 1998).

We performed qRT‐PCR on an ABI 7500 Real‐Time PCR system (Applied Biosystems, Foster City, USA) and prepared samples such that all genes for the same samples were run on the same plate, in duplo. The PCR program, quality control, and calculation of relative expression are explained in Appendix Note S1. We normalized relative expression to absolute feeding damage through dividing it by the damaged area in mm^2^.

### 2.8 Statistics

We used R v3.2.4 (R Core Team, 2016) for all statistical analyses. First, to investigate variation among *T. evansi* populations for feeding damage, GUS activity and relative transcript abundance of tomato genes, we defined statistical models with *T. evansi* population (categorical, 11 levels) and plant stage (categorical, 2 levels) as fixed factors, and experimental block (categorical, 5 levels) as a random factor. Because the response variables are on a continuous scale, we assumed Gaussian error distributions and implemented these models using package *lme4* (Bates, Mächler, Bolker, & Walker, 2015). We square root‐transformed GUS activity and relative transcript abundance to meet assumptions of normality, homogeneity of variance, independence and absence of negative fitted values. We assessed the significance of the *T. evansi* population factor using approximate *F* tests with a Kenward–Roger approximation as implemented in the package *pbkrtest* (Halekoh & Højsgaard, 2014). This approximation estimates the denominator degrees of freedom in the *F* test, producing decimal values. We calculated pairwise post hoc contrasts between treatments using the package *multcomp* (Hothorn, Bretz, & Westfall, 2008) and corrected for multiple testing with Holm's method.

Because our main purpose was to investigate variation in defense suppression among *T. evansi* populations, in all figures we report the results of tests from which the treatment with the defense‐inducing *T. urticae* had been excluded. However, to assess if tomato responses were more induced in the *T. urticae* treatment than in the *T. evansi* treatments, as a verification of defense suppression, we separately analyzed models where *T. urticae* was included as a treatment and report their outcomes in the text of the Results section. In addition, as explained above, we normalized GUS activity and qRT‐PCR results to differences in feeding damage among samples, precluding comparisons with uninfested control treatments. However, to assess if tomato defense expression differed between infested and uninfested treatments, we also analyzed models where GUS activity and relative transcript abundance of defense‐associated tomato genes had not been normalized, and report their outcomes in Figures S2 and S3.

Next, to explore which factors correlate with variation in tomato defense expression, we defined models with damage‐corrected relative transcript abundance as a response variable (continuous), experimental block (categorical, 5 levels), and *T. evansi* population (categorical, 11 levels) as crossed random factors, and either range (categorical, 2 levels), laboratory host plant (categorical, 2 levels), field host plant (categorical, 3 levels), or time in culture (continuous) as a fixed factor, as well as plant stage (categorical, 2 levels). We expressed the time that populations had been cultured in laboratory environments in an estimated number of generations, assuming a generation time of 14 days at 25°C (Bonato, 1999). We square root‐transformed relative transcript abundance to meet model assumptions and assessed the significance of terms using approximate *F* tests with a Kenward–Roger approximation.

### 2.9 CO1 sequencing

To determine the genetic lineage (Boubou et al., 2012; Gotoh et al., 2009) to which the *T. evansi* populations used in this study belong, we sequenced a part of the mitochondrial CO1 gene (Appendix Note S2). CO1 sequences were deposited in GenBank under accession numbers MT019694–MT019820 (Table S2).

### 2.10 Phylogeny construction

We edited, assembled, and aligned DNA sequences (900 bp) in Codoncode Aligner (version 5.0.2, Codoncode Corporation, Dedham, US). We removed primers and low‐quality reads and verified our contigs by using nucleotide blasts (National Centre of Biotechnology Information, US, http://blast.ncbi.nlm.nih.gov/Blast.cgi) after which we clipped them to remove gaps at terminal sites and realigned them in MEGA version 7.0.25 (Kumar, Stecher, & Tamura, 2015) using MUSCLE (Edgar, 2004). This alignment consisted of 127 sequences (868 bp), plus 7 reference sequences from GenBank: a *CO1* sequence of *T. urticae* (accession number: NC_010526, Van Leeuwen et al., 2008) as an outgroup and six *T. evansi CO1* sequences (accession numbers: FJ440675, FJ440676, FJ440677 and FJ440678 (Gotoh et al., 2009) and KF447575 and KF447576 (Alba et al., 2015)). We then used jModelTest version 2.1.10 (Darriba, Taboada, Doallo, & Posada, 2012) to select the general time‐reversible model (Tavaré, 1986) with substitution rate variation among sites (GTR + G, gamma shape = 0.2376) as the optimal nucleotide substitution model and constructed a maximum likelihood phylogenetic tree with 5,000 bootstraps using MEGA (Hall, 2013).

## 3 RESULTS

To investigate variation in defense suppression among the *T. evansi* populations, we first quantified differences in feeding damage and assessed the magnitude of the JA‐responses via measuring GUS activity in *pLAP‐1A:GUS* plants. We found significantly different amounts of damage (7–28 mm^2^ of leaf tissue per leaflet) among populations (*F*_10,112_ = 3.99, *p* < .001, Figure S1). When normalized for feeding damage, GUS activity was highly variable but not significantly different among populations (*F*_10,112_ = 0.78, *p* = .644, Figure S2). We also observed low levels of fluorescence in some of the control treatments (Figure S2B), which could be an indication of enzymatic activity in the absence of GUS. Possibly, the activity of tomato glycosidases other than GUS introduced some background variability in our measurements (Gu, Pautot, Holzer, & Walling, 1996).

**Figure 2:**
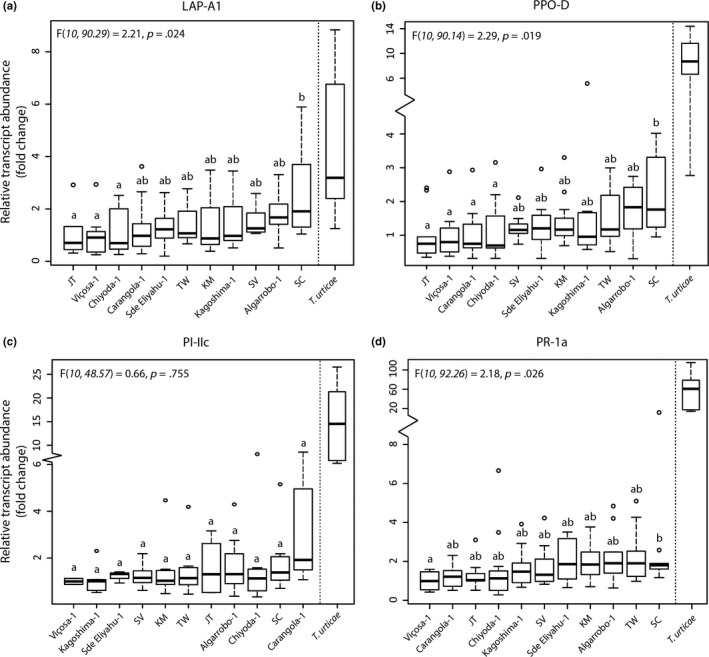
Expression of the plant defense‐associated marker genes *LAP‐A1* (a), *PPO‐D* (b), *PI‐IIc* (c), and *PR‐1a* (d) in *LAP:GUS* tomato plants after 1 day of infestation with adult *Tetranychus evansi* or *T. urticae* females from different populations. Gene expression was measured using qRT‐PCR and expressed in transcript abundance relative to that of *actin*, corrected for differences in feeding damage, and normalized to the lowest treatment mean. Details of statistical tests for differences among *T. evansi* populations are given in the upper left corners of each graph. Gene expression of plants infested by a defense‐inducing *T. urticae* population is shown on the right end of each graph, but was not included in statistical tests. Populations are ordered by increasing mean. This may change the order of populations among figures. Thick lines indicate treatment median, boxes encompass data from first to third quartile, whiskers indicate fences (nearest observed value ≥ first or ≤third quartile ± 1.5 box height), circles indicate outliers, and different letters indicate significant differences between treatments as assessed through Holm‐adjusted post hoc contrasts

To obtain more specific insight into the activation of tomato defenses due to feeding by our different *T. evansi* populations, we used qRT‐PCR analysis to investigate expression of the JA‐responsive defense marker genes *LAP‐A1*, *PPO‐D* and *PI‐IIc,* and the SA‐dependent gene *PR‐1a*. Except for *PI‐IIc,* we found significantly different expression of all three marker genes among tomatoes infested with the different *T. evansi* populations (Figure 2). Populations JT and Viçosa‐1 suppressed *LAP‐A1*, *PPO‐D,* and *PR‐1a* the strongest, whereas population SC allowed the strongest induction in tomato. These patterns did not correlate with differences in feeding damage, because Pearson correlations between damaged area and corrected relative transcript abundances were below 0.2 and nonsignificant (*p* > .1) for all defense marker genes.

The *T. urticae* genotype Santpoort‐2, our benchmark treatment for defense induction, induced higher expression than any of the *T. evansi* populations for all marker genes (all pairwise comparisons *p* < .05), except for *LAP‐A1* expression, which was similar between tomatoes infested with SC and Santpoort‐2 (*p* = 1.00). Although *T. evansi* was previously found to sometimes suppress tomato defense expression significantly below control levels (Godinho et al., 2016; de Oliveira, Pallini, & Janssen, 2016; Sarmento, Lemos, Bleeker, et al., 2011), we found expression levels to be similar to the levels in control plants or to be slightly higher for *PPO‐D*, *PI‐IIc,* and *PR‐1a* and to be significantly higher for *LAP‐A1* (Figure S3).

**Figure 3:**
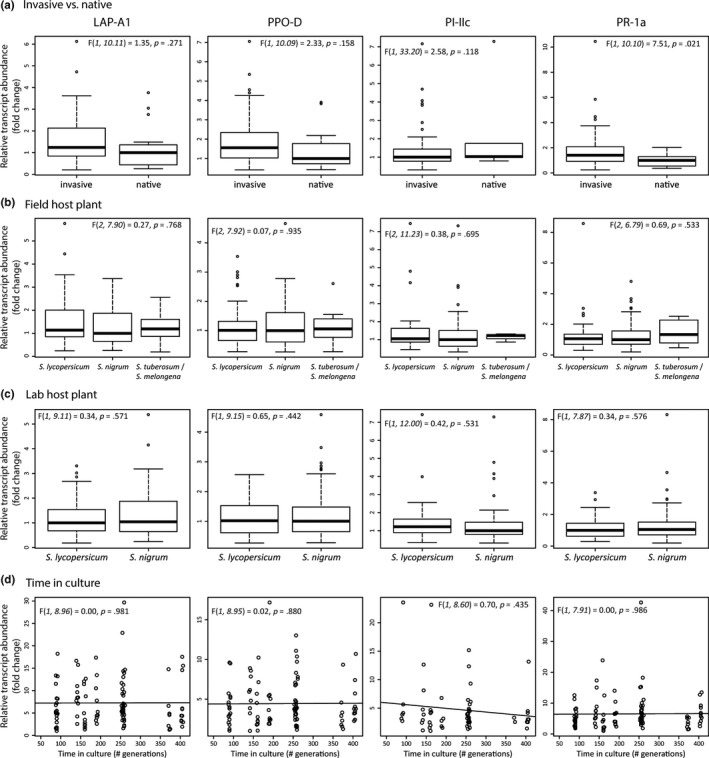
Variation in expression of defense‐associated tomato genes compared between native and invasive populations (a), among field host plant species (b), laboratory host plant species (c), and depending on the time the populations have been cultured in the laboratory (d). Gene expression of *LAP‐A1*, *PPO‐D*, *PI‐IIc,* and *PR‐1a* was measured using qRT‐PCR and expressed in transcript abundance relative to that of *actin* and corrected for differences in feeding damage. Details of statistical tests for differences in relative transcript abundance are given in the upper corners of each graph. In panels a–c, values were normalized to the lowest treatment median. Thick lines indicate treatment median, boxes encompass data from first to third quartile, whiskers indicate fences (nearest observed value ≥ first or ≤ third quartile ± 1.5 box height), and circles indicate outliers. In panel d, values were normalized to the smallest individual relative expression. Circles indicate data points and lines indicate linear model predictions for relative transcript abundance over time in culture

To further explore the observed variation in defense suppression among *T. evansi* populations, we assessed the correlation between marker gene expression levels and either geographical range, host plant, or time in culture. We found that invasive populations tended to suppress tomato defense less strongly than native populations, and this pattern was significant for the level of *PR‐1a* expression (Figure 3a). Expression levels did not correlate with the host plant species from which the *T. evansi* populations had been collected (Figure 3b). Likewise, expression levels were similar among plants infested with *T. evansi* populations cultured on *S. lycopersicum* or *S. nigrum* (Figure 3c) and did not correlate with the time that populations had been maintained in laboratory environments (Figure 3d).

**Figure 4:**
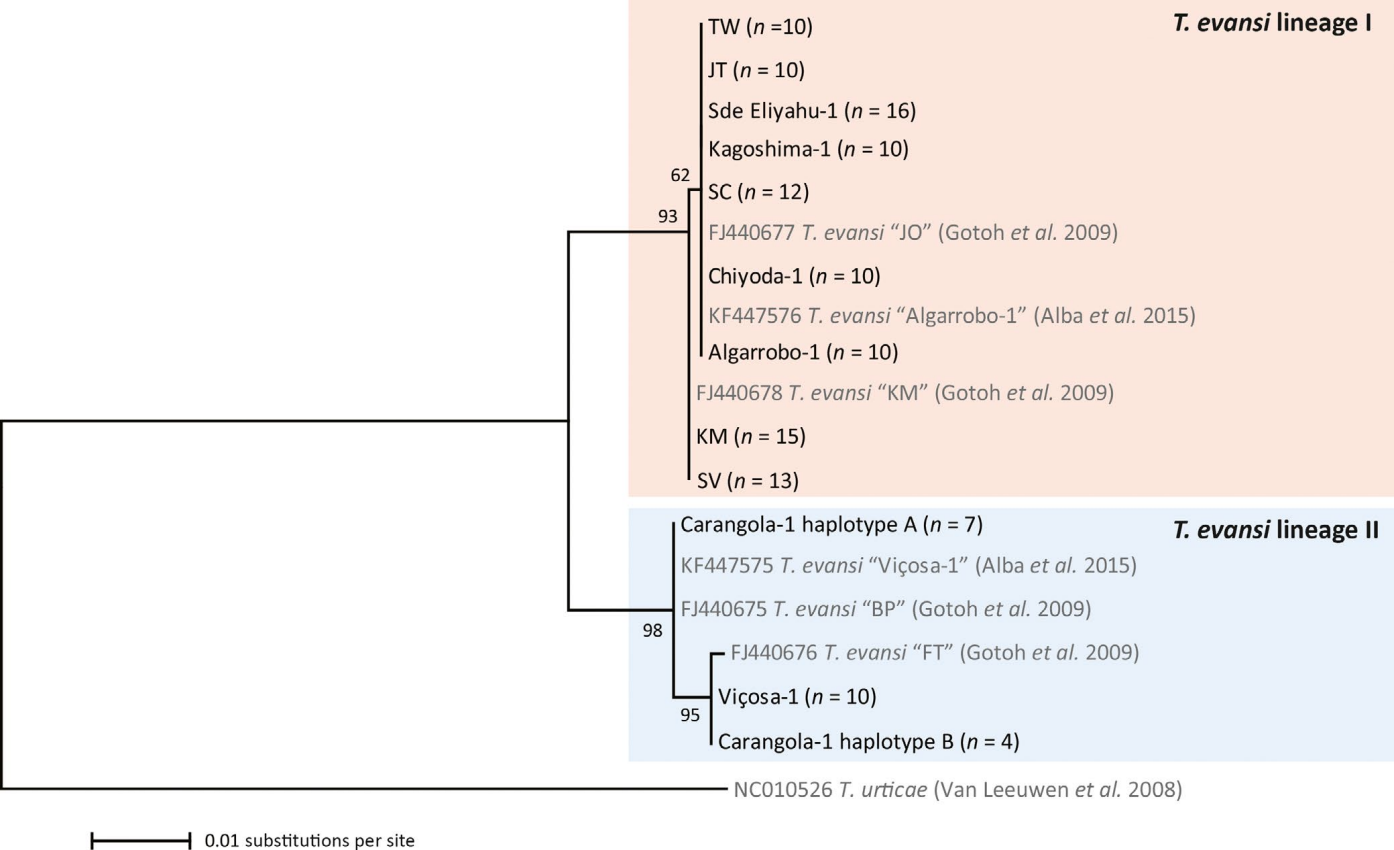
Phylogenetic relationships between *Tetranychus evansi* populations based on mitochondrial *CO1* gene sequences (868 bp). Relationships were inferred using the maximum likelihood method and the general time‐reversible model plus substitution rate variation among sites. Nucleotide positions with gaps or missing data (8.8%) were excluded. Branch support based on 5,000 bootstraps is indicated above each node. Populations of which *CO1* sequences were collected in this study are shown in black font along with their sample size, and reference sequences are indicated by their GenBank accession numbers and shown in gray. The naming of the two differentiated *T. evansi* lineages is as in Boubou et al. (2012)

To determine to which of the two genetically differentiated *T. evansi* lineages (Boubou et al., 2012; Gotoh et al., 2009) our populations belonged, we sequenced a part of the mitochondrial *CO1* gene and found that all invasive populations belonged to lineage I and all native populations to lineage II (Figure 4). Geographical range and genetic lineage are therefore completely collinear variables in our dataset, which precludes disentangling their effects on variation in defense suppression among *T. evansi* populations. Within lineage II, we found further differentiation within the Carangola‐1 population, and our samples from the Viçosa‐1 population belonged to a different haplotype than previously archived *CO1* sequences from the same population (KF447575, Alba et al., 2015).

## 4 DISCUSSION

Multiple arthropod herbivore species suppress the defenses of their host plants to prevent exposure to harmful plant defense and enhance herbivore performance (Kant et al., 2015; Musser et al., 2002). Lowered plant defenses, however, may also increase the performance of competing herbivores and promote predation (Ataide et al., 2016; Glas et al., 2014; Kant et al., 2008; Sarmento, Lemos, Bleeker, et al., 2011; Schimmel et al., 2018; Schimmel, Ataide, Chafi, et al., 2017; Schimmel, Ataide, & Kant, 2017). Biotic interactions among defense‐suppressing herbivores and competitors or predators may therefore give rise to ecological costs associated with defense suppression and may vary among locations. To understand the role of biotic interactions in the evolution of defense suppression, it is necessary to quantify variation in defense suppression across different biotic environments.

The purpose of this study was to investigate intraspecific variation in defense suppression among *T. evansi* populations from eleven locations and secondarily to explore if suppression differed across host plant species and native or non‐native ranges. We found significant variation in expression of the JA‐responsive marker genes *LAP‐A1* and *PPO‐D*, and at the SA‐dependent locus *PR‐1a* (Figure 2). This shows that *T. evansi* populations suppress the two hormonal signaling pathways that regulate tomato defense expression against spider mites to varying degrees. Although the effect size of this variation was small relative to the magnitude of induction by the *T. urticae* genotype Santpoort‐2, small differences in defense gene expression still can correlate with significant differences in spider mite performance (Alba et al., 2015). For example, low levels of JA‐dependent defense induction reduce *T. evansi* performance considerably, but stronger induction does not reduce *T. evansi* fecundity any further (Ataide et al., 2016). Because the observed variation in the level to which tomato defenses are suppressed by our *T. evansi* populations likely falls within this lower range of tomato defense induction, these differences can have substantial consequences for *T. evansi* performance.

We assessed if the observed variation in defense suppression among *T. evansi* populations correlated with their geographical range or with the identity of their host plant species. We expected invasive populations to suppress plant defense more strongly than native populations, because the absence of natural enemies in invasive populations alleviates ecological costs, such as increased predation by *P. longipes* predatory mites (Ataide et al., 2016). On the contrary, we found a trend that invasive *T. evansi* populations suppress tomato defenses less strongly in their invasive range than populations that are endemic to their habitat (Figure 3a). Possibly, *T. evansi* and *P. longipes* are engaged in an arms race (Dawkins & Krebs, 1979) over plant defense signaling. Under this scenario, *T. evansi* is selected to suppress tomato defense to prevent detection by *P. longipes*. Because *P. longipes* is absent in areas where *T. evansi* is invasive, *T. evansi* may evolve a lower degree of defense suppression through antagonistic pleiotropy (Cooper & Lenski, 2000), or it may erode through genetic drift (Halligan & Keightley, 2009). To obtain more insight into the effect of enemy release (Colautti, Ricciardi, Grigorovich, & MacIsaac, 2004; Jeffries & Lawton, 1984) on *T. evansi* defense suppression, future research could investigate which kind and which amounts of volatiles *P. longipes* needs to detect *T. evansi*‐infested tomato plants (Sarmento, Lemos, Bleeker, et al., 2011). Insight into how suppression of plant defense affects the recruitment and performance of other natural enemies, such as *N. floridiana* fungi (Elliot et al., 2000; Hountondji, Sabelis, Hanna, & Janssen, 2005), also awaits further study.

Geographical range and genetic lineage are completely collinear variables in our dataset, and we cannot disentangle their effects on variation in defense suppression among *T. evansi* populations. Although morphologically similar (Gotoh et al., 2009), the two *T. evansi* lineages are partly reproductively isolated (Gotoh et al., 2009; Knegt et al., 2017). Differentiation between these lineages likely preceded invasion of areas outside South America, but among the invasive populations lineage I is more prevalent than lineage II (Boubou et al., 2012; Meynard, Migeon, & Navajas, 2013). Previous studies have found that lineage I tolerates colder temperatures than lineage II (Migeon, Auger, Hufbauer, & Navajas, 2015) and has higher expression of digestive proteases (Santamaría et al., 2018). Our results complement these findings by showing that lineage II tends to suppress tomato defenses more strongly than lineage I (Figure 3a), as all our invasive *T. evansi* populations belonged to lineage I and both native populations to lineage II. Therefore, an equally possible explanation for the observed trend in defense suppression between native and invasive populations is that already in South America differences among the habitats of the two *T. evansi* lineages, such as different abundance of competitors and predators, selected for different levels of defense suppression. Future work could confirm this hypothesis by characterizing more *T. evansi* populations from their native South American habitats.

The four *Solanum* host plant species used in this study vary in their defensive metabolites and may therefore harbor different arthropod communities (Cipollini & Levey, 1997; Girard et al., 2007; Hartl et al., 2010; Jared et al., 2016; Nohara et al., 2007; Tingey, 1984). Because the costs of defense suppression by *T. evansi* depend on biotic interactions with competitors and predators in these communities, we hypothesized that this variation could select *T. evansi* to suppress plant defenses to different degrees. However, we found no indication that host plant species identity explained variation in defense suppression among *T. evansi* populations (Figure 3b). Some of our mite populations were maintained on a different host plant than tomato, and these mites were therefore confronted with a new host plant during our experimental assay. In theory, this could cause extra effects on gene expression in the tomato plant other than due to differences in damage; however, we do account for the factor “host plant” in our statistical analysis. Therefore, the significant differences we find are supported even when effects of placing mites on a novel host plant were present. Future work could aim to characterize arthropod communities on these host plants in nature, to be able to assess their interactions with *T. evansi* and their potential effects on defense suppression in more detail.

The tomato genes assayed in this study constitute marker genes of tomato defense induction. This does not imply direct causal relationships between their gene products and spider mite performance. Although expression of PI genes and PI activity, for example, increase upon infestation with defense‐inducing *T. urticae* (Ataide et al., 2016; Godinho et al., 2016; de Oliveira et al., 2016; Sarmento, Lemos, Bleeker, et al., 2011), and a weak negative correlation between PI activity and *T. urticae* (but not *T. evansi*) performance was observed (de Oliveira et al., 2016), the efficiency of these compounds as digestive inhibitors has been questioned because spider mite guts may lack their enzymatic targets (Arnaiz et al., 2018; Santamaría et al., 2012). Similarly, plant PPOs have been hypothesized to react with plant phenolic compounds in the herbivore gut after ingestion to produce quinones, which subsequently damage enzymes, membranes, and DNA (Constabel & Barbehenn, 2008), thus decreasing herbivore performance. However, because these processes might not be effective in spider mite guts due to their acidity (Erban & Hubert, 2010; Martel et al., 2015), the defensive role of PPOs against spider mites also awaits experimental confirmation. Since it is not known which tomato genes have a causal relationship with spider mite performance, these defense marker genes may paint an incomplete quantitative picture, and possibly we overlook relevant defenses with different induction and suppression kinetics. It would for example be interesting to also investigate the accumulation of steroidal glycoalkaloids, since these correlate with resistance of nightshades to *T. evansi* (Jared et al., 2016). Nevertheless, because *T. evansi* was previously shown to be sensitive to the magnitude of JA‐defenses (Ataide et al., 2016), while *PPO‐D* and *PI‐IIc* have been shown to be reliable markers for the magnitude of this defense (Alba et al., 2015), our results must be largely relevant.

Suppression of plant defense by herbivorous arthropods is an intriguing phenomenon due to its complex ecological consequences (Kant et al., 2015). Biotic interactions with competitors and natural enemies may shape the costs associated with defense suppression (Ataide et al., 2016; Glas et al., 2014; Sarmento, Lemos, Bleeker, et al., 2011; Sarmento, Lemos, Dias, et al., 2011), and we found variation in defense suppression among *T. evansi* populations from various locations, potentially related to their varying biotic environments. Notably, however, *T. evansi* is not helpless against biotic threats. In response to the presence of competing *T. urticae* mites, *T. evansi* increases its web production to secure feeding sites (Sarmento, Lemos, Dias, et al., 2011) and increases fecundity to promote population growth (Schimmel, Ataide, Chafi, et al., 2017). Additionally, *T. evansi* males actively interfere with the reproduction of *T. urticae* females (Clemente, Rodrigues, Ponce, Varela, & Magalhães, 2016; Clemente et al., 2018; Sato, Alba, & Sabelis, 2014; Sato, Staudacher, & Sabelis, 2016). Moreover, in the presence of cues associated with *P. longipes*, *T. evansi* females choose to more often oviposit in their web, where their eggs are less prone to predation by *P. longipes* than on the leaf surface (Lemos et al., 2010). Although these traits may also entail costs (e.g., web production), they provide protection against competitors and natural enemies and thus “buffer” (Frank, 2007) *T. evansi* against the negative biotic consequences of defense suppression (Blaazer et al., 2018). In this context, future research could investigate if the degree to which *T. evansi* populations engage into such buffering behavior correlates with the variation in defense suppression observed in this study, because this may point toward ecological costs of defense suppression.

## CONFLICT OF INTEREST

None declared.

## AUTHOR CONTRIBUTIONS

All authors designed the research. BK and TTM: experimentation. BK and TTM: analysis of the data. BK: writing—the manuscript. All authors contributed to and approved the final version of the manuscript.

## Supporting information

Fig S1

Fig S2

Fig S3

Table S1‐S2

## Data Availability

Data are available at Dryad under https://doi.org/10.5061/dryad.np5hqbzpw (Knegt, Meijer, Kant, Kiers, & Egas, 2020). *CO1* sequences are available at GenBank (https://www.ncbi.nlm.nih.gov/) under accession numbers MT019694–MT019820.
