# Supplementary figures and images for: Tetranychus evansi spider mite populations suppress tomato defenses to varying degrees
Source: Ecol Evol. 2020 Apr 12;10(10):4375–90. doi: 10.1002/ece3.6204 (PMC7246200; doi:10.1002/ece3.6204)

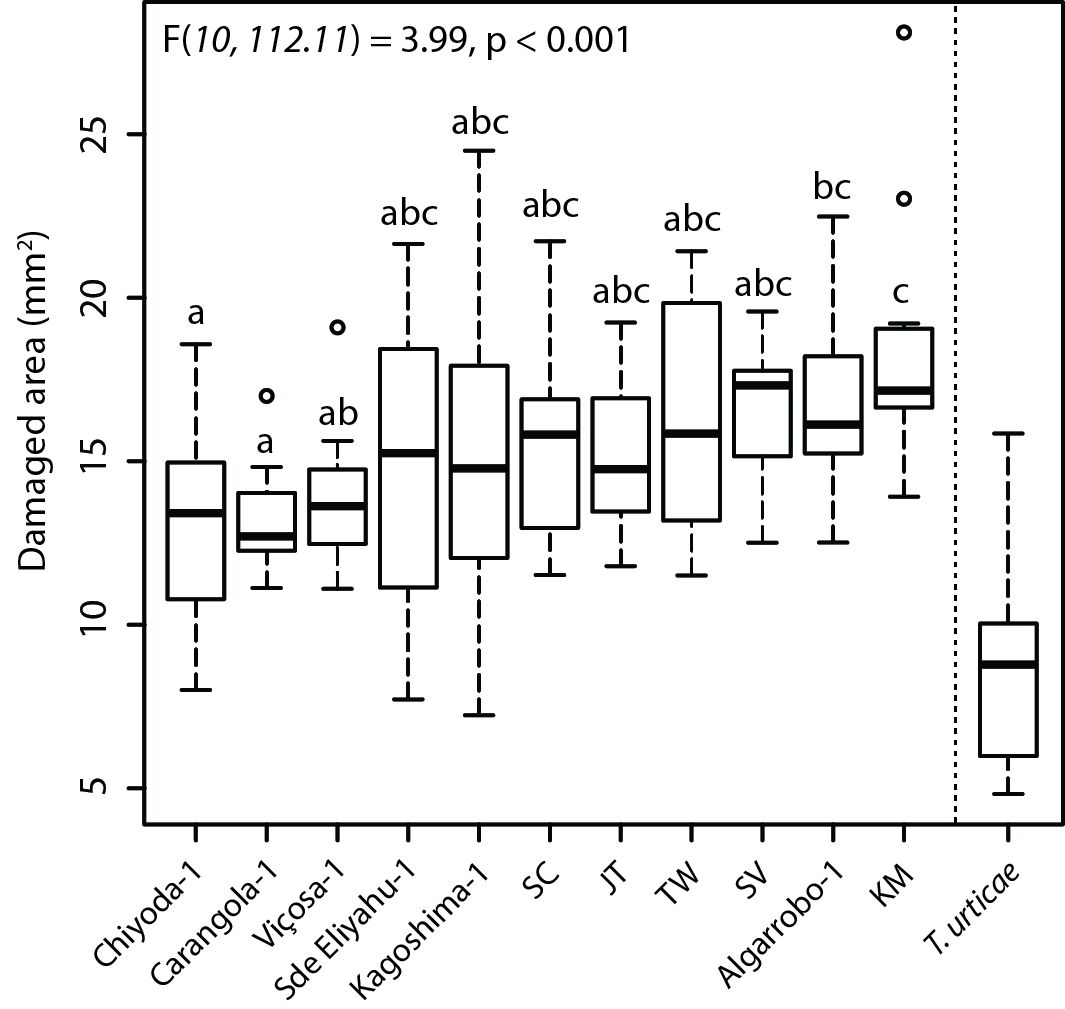

Supplement: Supplementary file 1 — Fig S1 [file ECE3-10-4375-s001.tif]

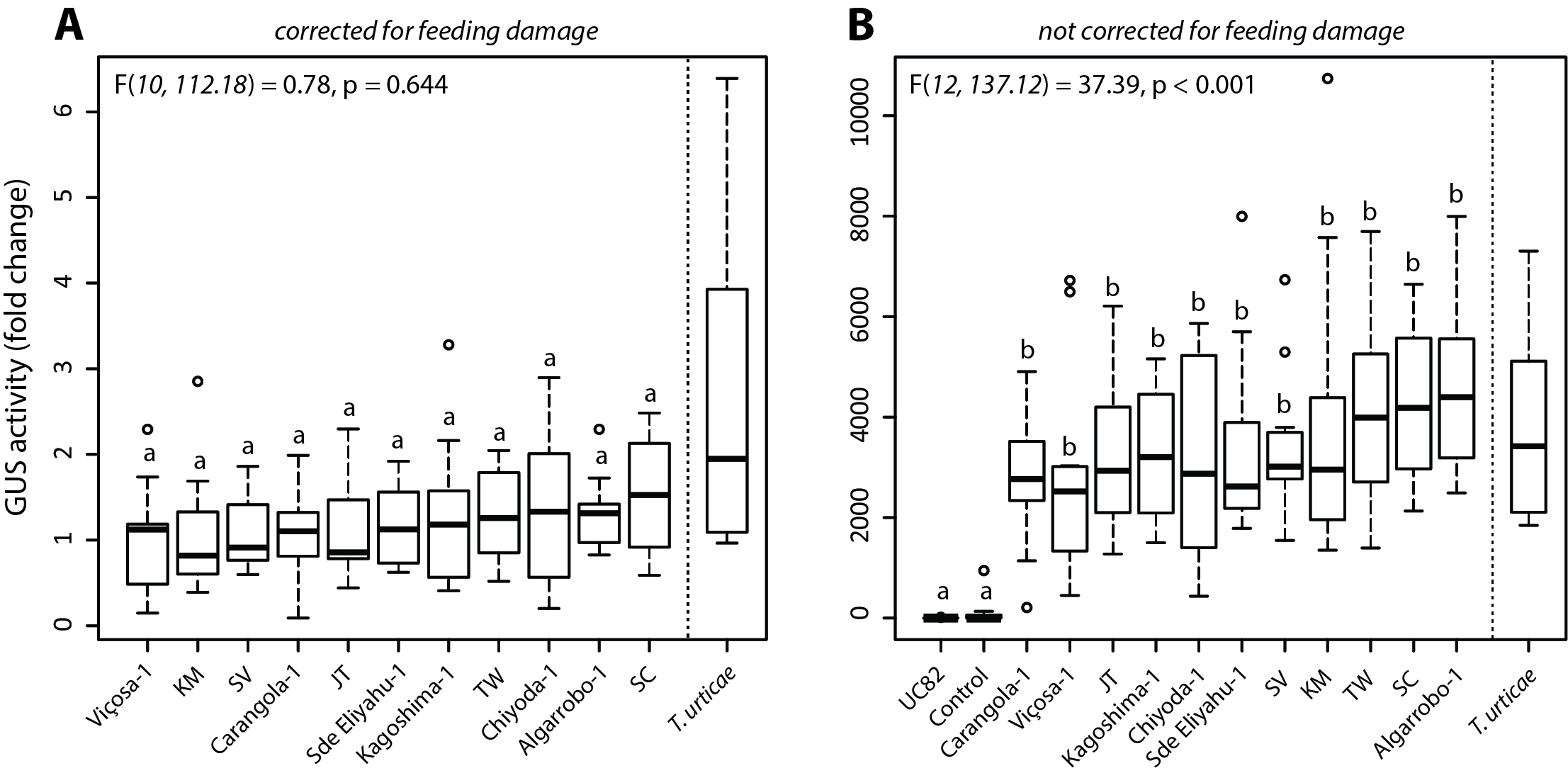

Supplement: Supplementary file 2 — Fig S2 [file ECE3-10-4375-s002.tif]

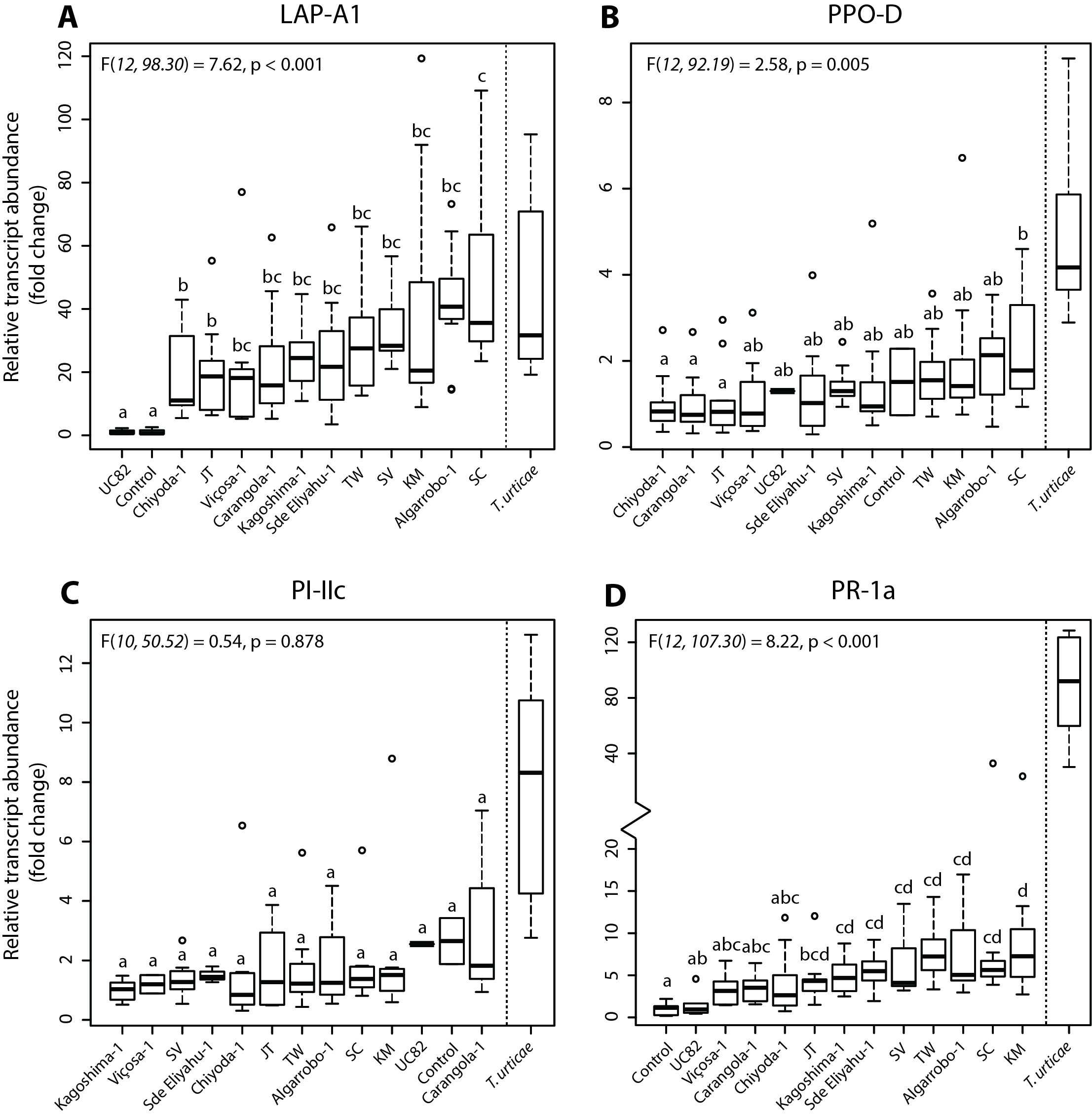

Supplement: Supplementary file 3 — Fig S3 [file ECE3-10-4375-s003.tif]
