# Supplementary material for: Tetranychus evansi spider mite populations suppress tomato defenses to varying degrees
Source: Ecol Evol. 2020 Apr 12;10(10):4375–90. doi: 10.1002/ece3.6204 (PMC7246200; doi:10.1002/ece3.6204)
Supplement: Supplementary file 4 — Table S1‐S2 [file ECE3-10-4375-s004.docx]

**Appendices**

Appendix Note S1. PCR program, quality control, and calculation of relative expression in qRT-PCR measurements.

We performed qRT-PCR on an ABI 7500 Real-Time PCR system (Applied Biosystems, Foster City, USA) in 20 μL reactions containing 7 μL cDNA solution, 4 μL EvaGreen (Solis BioDyne, Tartu, Estonia), 1 μL of both primers (5 μM, Table S1) and 7 μL H_2_0. We prepared samples such that all genes for the same samples were run on the same plate, in duplo, thus running 8 samples and 5 genes per plate. PCR conditions were initial heating at 50 °C for 2 min and 95 °C for 15 min, then 35 cycles of denaturation at 95 °C for 15 s and annealing / extension at 60 °C for 1 min, followed by a melt curve program. We inspected the amplification process of each individual reaction, and excluded reactions with a non-sigmoidal increase in fluorescence or with alternative melting curve peaks that were equally high or higher than the peak of the target amplicon (i.e., drops in fluorescence during melting curve analysis more than 2 °C different from the expected melting temperature of the target amplicon). These quality checks excluded 420 of 1336 (31%) reactions. We determined the reaction efficiency per target gene by taking the average of individual reaction efficiencies as calculated by LinRegPCR (Ruijter et al. 2009), while specifying a common threshold ^10^log fluorescence of 4.889 (average software-determined threshold across plates, standard error = 0.005) and a window-of-linearity per target gene. We then determined the cycle threshold values (C_t_-values) of each reaction by specifying the same threshold fluorescence, and averaged the two technical replicates into one C_t_-value per gene per sample, through taking $^{2}\log(\frac{2^{C_{t}(replicate 1)}+2^{C_{t}(replicate 2)}}{2})$. For each sample, we used this C_t_-value to calculate the expression of each gene relative to the expression of the *actin* housekeeping gene, as $\frac{efficiency locus^{-C_{t}(locus)}}{efficiency actin^{-C_{t}(actin)}}$.

Appendix Note S2. CO1 sequencing.

We extracted spider mite DNA from individual adult females of each population according to Walsh et al. (1991), by placing them in 100 μL 5% Chelex solution (Chelex 100 sodium form, Sigma-Aldrich, S*T.*Louis, USA) together with four zirconium beads, disrupting their tissues in a Precellys 24 homogenizer (Bertin Technologies, Montigny-le-Bretonneux, France) at 6000 rpm for 30 s, adding 5 μL proteinase K (20 mg/mL) to remove any nucleases from the solution, and incubating the samples at 56 °C for 60 min followed by denaturation at 95 °C for 8 min. Then, we amplified a part of the *CO1* gene through PCR in 25 μL solutions (13.5 μL H_2_O, 2.5 μL 10x PCR buffer [H T Biotechnology, Cambridge, UK], 2.5 μL dNTPs [1 mM each], 1.2 μL bovine serum albumin [Sigma-Aldrich, St*.*Louis, USA], 0.5 μL of both primers [10 μM, Table S1], 0.3 μL Taq polymerase, home-made according to Pluthero [1993], and 4 μL DNA extract), through initial denaturation at 94 °C for 2 min, 35 cycles of denaturation at 94 °C for 20 s, annealing at 50 °C for 30 sec, and extension at 72 °C for 55 s, followed by final extension at 72 °C for 10 min and cooling at 10 °C for 10 min, using a Bio-Rad T100 Thermal Cycler (Bio-Rad Laboratories, Hercules, USA). We confirmed PCR amplification on ethidium bromide-stained agarose gels, and then sent 10 μL solutions (1 μL PCR product, 1 μL primer (10 μM) and 8 μL H_2_O) for sequencing to the Macrogen EZ-seq service (Macrogen Europe, Amsterdam, Netherlands).

Appendix Table S1. Specifications of primers used for qRT-PCR and *CO1* sequencing.

| Target locus | Primer | Sequence (5’ -> 3’) | Reference |
| --- | --- | --- | --- |
| *actin* | forward | TTAGCACCTTCCAGCAGATGT | Tomato Genome Consortium (2012) |
|  | reverse | AACAGACAGGACACTCGCACT |  |
| *LAP-A1* | forward | ATCTCAGGTTTCCTGGTGGAAGGA | Fowler *et al.* (2009) |
|  | reverse | AGTTGCTATGGCAGAGGCAGAG |  |
| *PPO-D* | forward | GCCCAATGGAGCCATATC | Newman *et al.* (1993) |
|  | reverse | ACATTCGATCCACATTGCTG |  |
| *PI-IIc* | forward | CAGGATGTACGACGTGTTGC | Gadea *et al.* (1996) |
|  | reverse | GAGTTTGCAACCCTCTCCTG |  |
| *PR-1a* | forward | TGGTGGTTCATTTCTTGCAACTAC | van Kan *et al.* (1992) |
|  | reverse | ATCAATCCGATCCACTTATCATTTTA |  |
| *CO1* | forward | GGAGGATTTGGAAATTGATTAGTTCC | Gotoh *et al.* (2009) |
|  | reverse | GATAAAACGTAATGAAAATGAGCTAC |  |

Appendix Table S2. GenBank Accession numbers of submitted *CO1* sequences.

| **Sample name** | **GenBank Accession number** |
| --- | --- |
| CO1_Algarrobo-1_Sample_1 | MT019694 |
| CO1_Algarrobo-1_Sample_3 | MT019695 |
| CO1_Algarrobo-1_Sample_4 | MT019696 |
| CO1_Algarrobo-1_Sample_5 | MT019697 |
| CO1_Algarrobo-1_Sample_6 | MT019698 |
| CO1_Algarrobo-1_Sample_7 | MT019699 |
| CO1_Algarrobo-1_Sample_8 | MT019700 |
| CO1_Algarrobo-1_Sample_9 | MT019701 |
| CO1_Chiyoda-1_Sample_1 | MT019702 |
| CO1_Chiyoda-1_Sample_10 | MT019703 |
| CO1_Chiyoda-1_Sample_2 | MT019704 |
| CO1_Chiyoda-1_Sample_3 | MT019705 |
| CO1_Chiyoda-1_Sample_4 | MT019706 |
| CO1_Chiyoda-1_Sample_5 | MT019707 |
| CO1_Chiyoda-1_Sample_6 | MT019708 |
| CO1_Chiyoda-1_Sample_7 | MT019709 |
| CO1_Chiyoda-1_Sample_8 | MT019710 |
| CO1_Chiyoda-1_Sample_9 | MT019711 |
| CO1_JT_Sample_1 | MT019712 |
| CO1_JT_Sample_2 | MT019713 |
| CO1_JT_Sample_3 | MT019714 |
| CO1_JT_Sample_4 | MT019715 |
| CO1_JT_Sample_5 | MT019716 |
| CO1_JT_Sample_6 | MT019717 |
| CO1_JT_Sample_7 | MT019718 |
| CO1_JT_Sample_9 | MT019719 |
| CO1_Kagoshima-1_Sample_1 | MT019720 |
| CO1_Kagoshima-1_Sample_10 | MT019721 |
| CO1_Kagoshima-1_Sample_2 | MT019722 |
| CO1_Kagoshima-1_Sample_3 | MT019723 |
| CO1_Kagoshima-1_Sample_4 | MT019724 |
| CO1_Kagoshima-1_Sample_5 | MT019725 |
| CO1_Kagoshima-1_Sample_6 | MT019726 |
| CO1_Kagoshima-1_Sample_7 | MT019727 |
| CO1_Kagoshima-1_Sample_8 | MT019728 |
| CO1_Kagoshima-1_Sample_9 | MT019729 |
| CO1_SC_Sample_1 | MT019730 |
| CO1_SC_Sample_10 | MT019731 |
| CO1_SC_Sample_11 | MT019732 |
| CO1_SC_Sample_12 | MT019733 |
| CO1_SC_Sample_2 | MT019734 |
| CO1_SC_Sample_3 | MT019735 |
| CO1_SC_Sample_4 | MT019736 |
| CO1_SC_Sample_5 | MT019737 |
| CO1_SC_Sample_6 | MT019738 |
| CO1_SC_Sample_8 | MT019739 |
| CO1_SC_Sample_9 | MT019740 |
| CO1_Sde_Eliyahu-1_Sample_1 | MT019741 |
| CO1_Sde_Eliyahu-1_Sample_10 | MT019742 |
| CO1_Sde_Eliyahu-1_Sample_11 | MT019743 |
| CO1_Sde_Eliyahu-1_Sample_13 | MT019744 |
| CO1_Sde_Eliyahu-1_Sample_2 | MT019745 |
| CO1_Sde_Eliyahu-1_Sample_3 | MT019746 |
| CO1_Sde_Eliyahu-1_Sample_4 | MT019747 |
| CO1_Sde_Eliyahu-1_Sample_5 | MT019748 |
| CO1_Sde_Eliyahu-1_Sample_6 | MT019749 |
| CO1_Sde_Eliyahu-1_Sample_7 | MT019750 |
| CO1_Sde_Eliyahu-1_Sample_8 | MT019751 |
| CO1_Sde_Eliyahu-1_Sample_9 | MT019752 |
| CO1_Algarrobo-1_Sample_10 | MT019753 |
| CO1_Algarrobo-1_Sample_2 | MT019754 |
| CO1_TW_Sample_1 | MT019755 |
| CO1_TW_Sample_10 | MT019756 |
| CO1_TW_Sample_2 | MT019757 |
| CO1_TW_Sample_3 | MT019758 |
| CO1_TW_Sample_4 | MT019759 |
| CO1_TW_Sample_5 | MT019760 |
| CO1_TW_Sample_6 | MT019761 |
| CO1_TW_Sample_7 | MT019762 |
| CO1_TW_Sample_8 | MT019763 |
| CO1_TW_Sample_9 | MT019764 |
| CO1_KM_Sample_5 | MT019765 |
| CO1_KM_Sample_1 | MT019766 |
| CO1_KM_Sample_13 | MT019767 |
| CO1_KM_Sample_15 | MT019768 |
| CO1_KM_Sample_2 | MT019769 |
| CO1_KM_Sample_3 | MT019770 |
| CO1_KM_Sample_4 | MT019771 |
| CO1_KM_Sample_8 | MT019772 |
| CO1_KM_Sample_9 | MT019773 |
| CO1_SV_Sample_10 | MT019774 |
| CO1_SV_Sample_3 | MT019775 |
| CO1_SV_Sample_5 | MT019776 |
| CO1_SV_Sample_6 | MT019777 |
| CO1_JT_Sample_10 | MT019778 |
| CO1_Sde_Eliyahu-1_Sample_12 | MT019779 |
| CO1_Sde_Eliyahu-1_Sample_14 | MT019780 |
| CO1_Sde_Eliyahu-1_Sample_15 | MT019781 |
| CO1_KM_Sample_10 | MT019782 |
| CO1_KM_Sample_11 | MT019783 |
| CO1_KM_Sample_12 | MT019784 |
| CO1_KM_Sample_14 | MT019785 |
| CO1_KM_Sample_6 | MT019786 |
| CO1_SV_Sample_11 | MT019787 |
| CO1_SV_Sample_13 | MT019788 |
| CO1_Sde_Eliyahu-1_Sample_16 | MT019789 |
| CO1_SV_Sample_12 | MT019790 |
| CO1_SV_Sample_4 | MT019791 |
| CO1_SC_Sample_7 | MT019792 |
| CO1_JT_Sample_8 | MT019793 |
| CO1_SV_Sample_1 | MT019794 |
| CO1_SV_Sample_7 | MT019795 |
| CO1_KM_Sample_7 | MT019796 |
| CO1_SV_Sample_9 | MT019797 |
| CO1_SV_Sample_2 | MT019798 |
| CO1_SV_Sample_8 | MT019799 |
| CO1_Carangola-1_Sample_1 | MT019800 |
| CO1_Carangola-1_Sample_8 | MT019801 |
| CO1_Vicosa1_Sample_1 | MT019802 |
| CO1_Vicosa-1_Sample_10 | MT019803 |
| CO1_Vicosa-1_Sample_2 | MT019804 |
| CO1_Vicosa-1_Sample_3 | MT019805 |
| CO1_Vicosa-1_Sample_5 | MT019806 |
| CO1_Vicosa-1_Sample_6 | MT019807 |
| CO1_Vicosa-1_Sample_7 | MT019808 |
| CO1_Vicosa-1_Sample_8 | MT019809 |
| CO1_Vicosa-1_Sample_9 | MT019810 |
| CO1_Carangola-1_Sample_10 | MT019811 |
| CO1_Carangola-1_Sample_3 | MT019812 |
| CO1_Vicosa-1_Sample_4 | MT019813 |
| CO1_Carangola-1_Sample_11 | MT019814 |
| CO1_Carangola-1_Sample_2 | MT019815 |
| CO1_Carangola-1_Sample_5 | MT019816 |
| CO1_Carangola-1_Sample_6 | MT019817 |
| CO1_Carangola-1_Sample_7 | MT019818 |
| CO1_Carangola-1_Sample_9 | MT019819 |
| CO1_Carangola-1_Sample_4 | MT019820 |

Appendix Figure S1. Leaf area damaged by *T. evansi* or *T. urticae* spider mites from different populations after 1 day of feeding. Details of a statistical test for differences among *T. evansi* populations are given in the upper left corner. Leaf area damaged by the defense-inducing *T. urticae* population is shown on the right but was not included in the statistical test. Populations are ordered by increasing mean. This may change the order of populations among figures. Thick lines indicate treatment median, boxes encompass data from first to third quartile, whiskers indicate fences (nearest observed value ≥ first or ≤ third quartile ± 1.5 box height), circles indicate outliers, and different letters indicate significant differences between treatments as assessed through Holm-adjusted post hoc contrasts.

Appendix Figure S2. GUS activity in *LAP-A1:GUS* tomato plants after 1 day of infestation with *T. evansi* or *T. urticae* spider mites from different populations. GUS activity was measured in a fluorimetric assay, corrected for the total amount of protein extracted, and normalized to the lowest treatment mean. In panel **A** GUS activity was also corrected for differences in feeding damage between samples, whereas in panel **B** this correction was not performed to allow comparisons to uninfested (“Control”) and uninfested, untransformed (“UC82”) treatments. Details of statistical tests for differences among treatments are given in the upper left corners of each graph. GUS activity in plants after infestation by a defense-inducing *T. urticae* population is shown on the right end of each graph, but was not included in the statistical test. Populations are ordered by increasing mean. This may change the order of populations among figures. Thick lines indicate treatment median, boxes encompass data from first to third quartile, whiskers indicate fences (nearest observed value ≥ first or ≤ third quartile ± 1.5 box height), circles indicate outliers, and different letters indicate significant differences between treatments as assessed through Holm-adjusted post hoc contrasts.

Appendix Figure S3. Expression of the plant defense-associated marker genes *LAP-A1* (A), *PPO-D* (B), *PI-IIc* (C), and *PR-1a* (D) in *LAP-A1:GUS* tomato plants after 1 day of infestation with adult *T. evansi* or *T. urticae* females from different populations. Gene expression was measured using qRT-PCR and expressed in transcript abundance relative to that of *actin* and normalized to the lowest treatment mean. These values were not corrected for differences in feeding damage among treatments, to allow comparisons to uninfested (“Control”) and uninfested, untransformed (“UC82”) treatments. Details of statistical tests for differences among *T. evansi* populations are given in the upper left corners of each graph. Gene expression of plants infested by a defense-inducing *T. urticae* population is shown on the right end of each graph, but was not included in statistical tests. Populations are ordered by increasing mean. This may change the order of populations among figures. Thick lines indicate treatment median, boxes encompass data from first to third quartile, whiskers indicate fences (nearest observed value ≥ first or ≤ third quartile ± 1.5 box height), circles indicate outliers, and different letters indicate significant differences between treatments as assessed through Holm-adjusted post hoc contrasts.
